# Classification of Pediatric Asthma: From Phenotype Discovery to Clinical Practice

**DOI:** 10.3389/fped.2018.00258

**Published:** 2018-09-20

**Authors:** Ceyda Oksel, Sadia Haider, Sara Fontanella, Clement Frainay, Adnan Custovic

**Affiliations:** ^1^Section of Paediatrics, Department of Medicine, Imperial College London, London, United Kingdom; ^2^Department of Epidemiology and Biostatistics, Faculty of Medicine, School of Public Health, Imperial College London, London, United Kingdom; ^3^INRA, UMR1331, Toxalim, Research Centre in Food Toxicology, Toulouse, France

**Keywords:** asthma, phenotypes, disease progression, machine learning, longitudinal data, big data

## Abstract

Advances in big data analytics have created an opportunity for a step change in unraveling mechanisms underlying the development of complex diseases such as asthma, providing valuable insights that drive better diagnostic decision-making in clinical practice, and opening up paths to individualized treatment plans. However, translating findings from data-driven analyses into meaningful insights and actionable solutions requires approaches and tools which move beyond mining and patterning longitudinal data. The purpose of this review is to summarize recent advances in phenotyping of asthma, to discuss key hurdles currently hampering the translation of phenotypic variation into mechanistic insights and clinical setting, and to suggest potential solutions that may address these limitations and accelerate moving discoveries into practice. In order to advance the field of phenotypic discovery, greater focus should be placed on investigating the extent of within-phenotype variation. We advocate a more cautious modeling approach by “supervising” the findings to delineate more precisely the characteristics of the individual trajectories assigned to each phenotype. Furthermore, it is important to employ different methods within a study to compare the stability of derived phenotypes, and to assess the immutability of individual assignments to phenotypes. If we are to make a step change toward precision (stratified or personalized) medicine and capitalize on the available big data assets, we have to develop genuine cross-disciplinary collaborations, wherein data scientists who turn data into information using algorithms and machine learning, team up with medical professionals who provide deep insights on specific subjects from a clinical perspective.

## Introduction

Asthma is a term describing a heterogeneous medical condition characterized by variable symptom expression, airway inflammation and therapeutic responses, making the clinical diagnosis challenging and long-term prognosis uncertain (1). Identifying genetic risk factors, environmental associates and pathophysiological mechanisms of asthma is further complicated by the fact that there is no uniform definition of this condition (2–4). In research settings, different studies use different definitions, which may lead to the under- or over-estimation of cases, and any signal in genetic of environmental association studies may be diluted as a consequence of the heterogeneity of the primary outcome measure (5). For example, Van Wonderen et al. reviewed 122 published articles and reported a staggering 60 different definitions of childhood asthma used in cohort studies (4). After selecting four common definitions used in the literature and applying them to a single cohort, the authors found that prevalence estimates varied from 15.1 to 51.1% (4). For the clinical setting, the UK National Institute of Health and Care Excellence (NICE) guidance recommends algorithm for diagnosing childhood asthma which is based on sequential assessment of four objective tests of lung function/airway inflammation (spirometry, bronchodilator reversibility, fractional exhaled nitric oxide, and peak flow variability; https://www.nice.org.uk/guidance). However, a recent study has found a poor agreement between the proposed algorithm and a strict epidemiological definition of asthma (physician diagnosis, current symptoms, and regular use of inhaled corticosteroids) in a birth cohort study (6). The authors suggested that the proposed NICE guidance on asthma diagnosis in children should not be implemented, emphasizing the uncertainties of how to accurately diagnose asthma, and which objective tests are useful (6).

There is increasing recognition that asthma is not a single disease, but a collective noun used to describe a set of clinical symptoms and features which may arise through different pathophysiological mechanisms (7, 8). While the subtypes of asthma sharing similar observable characteristics are often labeled as “phenotypes,” “asthma endotypes” are defined on the basis of pathophysiological mechanisms associated with discrete subtypes. There is a general consensus in the medical community that different endotypes of asthma do exist, however, there is no consensus as to what these endotypes are, or how to define them (9). One approach to endotype discovery capitalizes on the advances in computer sciences and software engineering and uses unbiased, data-driven approaches in an attempt to uncover different “phenotypes” of asthma, with the assumption that patterns of clinical symptoms are a reflection of specific underlying pathophysiological mechanisms (9). It is important to emphasize that disease subtypes discovered using data-driven approaches are not observed, but latent (i.e., hidden) by nature, and ideally should not be referred to as “phenotypes” (i.e., observable characteristics). However, as the term “phenotype” has been used in this context for more than a decade (10), we will maintain this nomenclature in this review. A thorough review of the implementation of data-driven methods for phenotype discovery in pediatric asthma has been conducted recently, with a particular focus on childhood wheezing illness and different “wheezing phenotypes” at a population level (11, 12). We will expand the discussion beyond the existing approaches to understanding phenotypic complexity in asthma, and highlight the role of clinical context and clinical experience in linking latent “phenotypes” to underlying biological mechanisms and tailored treatment approaches. We start by highlighting the heterogeneity of asthma and its phenotypic expression, and then discuss potential solutions to maximize the gain from different sources of data, and their clinical utility in asthma research.

## Disentangling asthma heterogeneity: from subjective to data-driven approaches

The idea of characterizing asthma subtypes based on the temporal pattern of symptoms through the life-course is not new (13), but has gained momentum in recent years with emerging of data-driven analytic approaches. Over the past two decades, subtyping approaches have progressed from subjective sub-typing to statistical classification techniques. Table 1 summarizes different approaches for discovering pediatric asthma phenotypes. In subjective sub-typing, phenotypes are identified using predefined or hypothesized criteria based on investigators' insights about clinical features, symptoms, age of onset, and progression rate (14, 23). The main limitation of this approach is that less obvious or rare patterns may be missed. A risk of artificially limiting the set of inputs or imposing a structure on the data is that it may limit the predictive ability of a model by missing associations which do, in fact, exist (9). In contrast, data-driven classification relies on techniques and algorithms that mine the large data sets to uncover the underlying structures and patterns “hidden” in the data. Statistical methods such as cluster analysis and latent class analysis (LCA) (11, 24–26), principal component analysis (20, 27), and exploratory factor analysis (21), have been widely applied to discover homogeneous subtypes of asthma. These procedures ranged from univariate approaches (a single symptom measured over time) to more sophisticated, multivariate approaches that simultaneously model several variables, including symptoms and other clinical and environmental characteristics. By incorporating the longitudinal structure of data, the latter has enabled investigators to capture the multidimensionality of the disease and to characterize phenotypic heterogeneity across the life-course (28).

**Table 1:** Different approaches for phenotypic discovery with the associated advantages and disadvantages.

**References**	**Age (years)**	**Sample size**	**Methodology**	**Strengths**	**Limitations**
(13)	1–6	54	Subjective sub-typing	- Phenotypes are observable expressions - Choice of cutoff guided by investigator expertise - Simple	- Predefined or hypothesized criteria needed - Rare patterns may be missed - Risk of over- or under- fitting as there are no objective statistical criteria for judging fit - Subjective cut-offs need to be recalibrated when new data becomes available - Un-validated cut-offs pose challenge for comparing findings across studies
(14)	1–6	826			
(15)	1–6	6265	Latent class analysis	- Probabilistic class allocation. - No prior knowledge is needed. - Hidden patterns may be uncovered that could not be a priori. - Hypothesis generating - Objective statistical criteria for judging whether phenotypes represent true variation	- Discovered sub-types are latent and retrospective by nature - Within-class heterogeneity arising from individuals whose patterns do not exemplify any phenotype - Meaningful clinical interpretation required to explain the patterns - Number of derived phenotypes may be related to the frequency and timing of data collection - Unclear to what extent established phenotype labels convey temporal patterns
(10)	1–7	689			
(16)	1–9	953			
(17)	1–8	5760			
	1–8	2810			
(18)	1–8	1184			
(19)	8–12	3890			
(20)	3–5	946	Principal component analysis	- Accounts for - coexisting symptoms - Reduces the variable dimensions in complex diseases	- Difficult clinical interpretation - Not useful for categorical and longitudinal data unless properly specified
(21)	7–35	925	Exploratory factor analysis		
(22)	6–18	613	Hierarchical clustering	- No a priori info about the number of classes required	- Risk of misclassifying distinct phenotypes that are present at low frequency

Nowadays, big data set containing many thousands of variables (such as clinical variables, objective tests, various biomarkers, genome-wide genotyping, proteomics etc.), are extensively used in medical research. In particular, the concept of “big” is difficult to pin down and relative to each field. Big data in healthcare refers to the large volumes of data accumulated from numerous sources, patients and populations that can no longer be easily handled by traditional statistical analysis methods due to its complexity. One of the advantages of big data in medicine is its capacity to examine heterogeneity between diverse populations, build better predictive models around individual patients and deliver more personalized and effective care. As an example, big data could be used to develop analytical tools that can help identify at-risk asthma patients before an attack occurs^1^, to identify patients with exacerbations and inadequately controlled asthma (29) and to understand how variations in environmental factors influence childhood asthma hospitalization (30).

**Figure 1:**
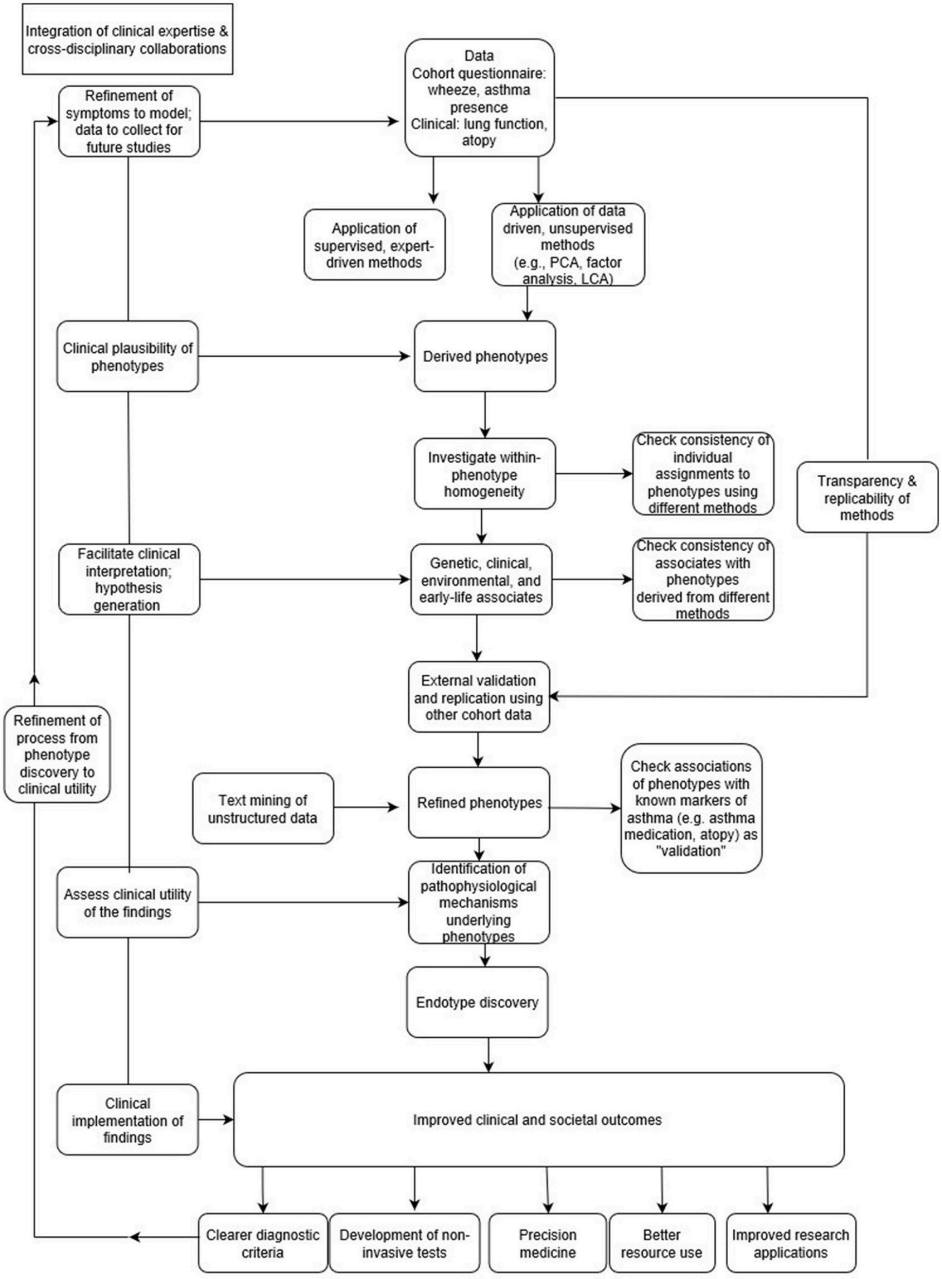
From phenotype discovery to clinical utility.

In the context of “big data analytics,” it is not possible to define *a priori* all possible causal and associational mechanisms (9). By allowing algorithms to model a large number of potential associations in an unsupervised way, patterns can be identified that could not have been predicted in advance, even by experts in the field. As such, data is allowed to speak for itself, often without relying on any prior knowledge. However, a danger of this approach is that it may become divorced from rigorous scientific scrutiny and meaningful clinical interpretation (9), since big data can only explain part of the picture (31). In the absence of guidance about the clinical plausibility of findings, there is a risk of identifying false positive associations as the number of relationships being tested increases (32). To be genuinely successful, the “data-driven” approach should encompass making decisions based on both data analysis and interpretation (Figure 1), which can only be achieved through a true synergy between the expertise in data science and clinical domain (22).

## Latent variable modeling paradigms for “phenotype” identification

One way to address the complexity of asthma is to derive asthma phenotypes that differentiate groups of patients presenting with similar combinations of symptoms, and to understand how biological factors shape each of these disease “phenotypes” (5). One such approach is latent class trajectory modeling, a class of probabilistic models in which repeated measurements of observable symptoms are modeled to identify homogeneous sub-populations within the larger heterogeneous population. Over the last few decades, latent modeling approaches [reviewed in (9, 11, 12)] have been extensively used to identify longitudinal trajectories of childhood wheeze (10, 17, 33, 34), atopy (34–37), and asthma (11, 12, 19), and to evaluate their associations with early life risk factors. For example, recent studies which used data from several population-based birth cohort studies have described four discrete trajectories of lung function from early childhood to young adulthood (38, 39), providing evidence that early life influences might be crucial not only for childhood asthma, but also for the pathogenesis of COPD in adulthood (28).

However, despite the increasing utilization of (and reliance on) latent class methods to stratify asthma and allergic diseases, there is a striking lack of enquiry into the extent of between-individual variation within the supposedly homogeneous “phenotypes.” Latent class methods use posterior probabilities which provide researchers with an objective basis for assigning individuals to classes (phenotypes) that best typify their pattern of symptom development. As these probabilities collectively measure specific individual's likelihood of belonging to each of the classes discovered by a model, a class (or “phenotype”) membership is not fixed, and all individuals are assigned a non-zero probability of belonging to each class. It is a common practice to then assign individuals to one of the latent classes according to the maximum posterior probability for an individual belonging to a particular class. Once classified in such way, the individuals are often considered as members of a single class, despite occasionally a considerable variations in posterior probabilities and marginal class assignments. However, in some cases, there may be subjects who have low posterior probabilities for all classes, and/or whose patterns do not exemplify any phenotype. As an example, an individual may have a 0.5 probability of belonging to “phenotype 1,” 0.30 probability of belonging to “phenotype 2,” and 0.20 probability of being in “phenotype 3,” yet such classification would assign a person into “phenotype 1,” ignoring the underlying uncertainty in class assignment. An implication of this is that the latent classes may not, in fact, reflect homogeneous patterns. If we are to understand pathophysiological processes underpinning different phenotypes, then each phenotype should include only individuals whose patterns fit well within the assigned class with a high probability (close to 1), and with a very low probability of belonging to other classes.

Furthermore, it is unclear to what extent the phenotypic nomenclature adequately conveys the temporal characteristics of individuals assigned to the classes, for example, whether “persistent” wheeze means long and/or uninterrupted spells of wheeze, and/or whether “early transient” means absolutely no recurrence of wheeze later in life. Also, one has to be careful not to assume that a persistence of symptoms (such as wheeze) necessarily reflects a persistence of the same pathophysiological process. For example, children with “persistent wheeze” may develop symptoms in early life due to impaired anti-virus responses (40), while the cause of the wheezing later in the school age may be related to other mechanisms such as IgE-mediated sensitisation (41). We would also like to highlight that phenotypes derived from different birth cohort studies often share the same nomenclature (such as “transient early,” “late-onset” and “persistent”), but phenotypes with the same assignment often differ substantially in terms of their age of onset, temporal trajectory and distributions within a population. Although common labels are frequently ascribed to latent classes (phenotypes) across studies, it has not been established whether individuals with similar longitudinal profiles are classified to the “same” phenotype in different cohorts sharing similar time points (or even within the same cohort). Moreover, classifications derived from latent class methods appear to be based on a combination of the timing of onset of symptoms and their frequency, but there has been a lack of research into whether there are different levels of disease “severity” within each phenotype, and how would the addition of information on severity impact classification.

A recent review of childhood wheeze phenotypes discovered using data-driven methods found a lack of consistent associations with risk factors and associates across different studies (18). We propose that within-class heterogeneity may be a in part responsible for these discrepancies, and may mask potentially important and consistent associations. Given that the optimal solution for the number of phenotypes may be an artifact of the underlying assumptions of the methods employed, idiosyncrasies particular to a cohort, and within-class heterogeneity, we encourage researchers to investigate the characteristics of the individual trajectories assigned to the phenotypes, and in doing so, question whether the model assumptions are appropriate for the data at hand. In order to reduce misclassification and derive more holistic phenotypes which reflect a” real life” and clinical practice, we would also suggest that rather than focusing on a single symptom (e.g., wheeze), we should employ methods that can incorporate a more comprehensive set of symptoms and/or comorbidities (for example, rhinitis, atopic dermatitis) (42). Thus, in order to achieve more consistency in phenotype discovery (in particular with respect to the role of different risk factors), it may be necessary to move beyond LCA and employ other methods for phenotype discovery.

## Advancing phenotype discovery: the case for a more refined approach

As outlined above, the identification of asthma phenotypes and their underlying distinct pathophysiological mechanisms is crucial for the development of targeted therapeutic strategies (1, 5, 8, 9). In order to achieve this goal, it is imperative that researchers derive asthma phenotypes that are truly homogenous. Whilst data-driven approaches have provided a framework for unearthing a structure within large datasets, there is a risk of assuming that the results represent the “truth,” in particular when this assumption is based on a reliance on objective statistical criteria, such as the Bayesian information criterion (BIC), Akaike information criterion (AIC), etc. For the clinical community, the proliferation of machine learning techniques and their associated language inventory of “new” terms [hidden Markov models (34), random forest (42), Bayesian networks (42), latent variable modeling (42), clustering (22), etc.,] are complex to comprehend, even by the statistically literate. Rigorous scientific assessment, reproducibility and transparency of models are increasingly challenging with the availability of diverse programming languages (R, Python, Stata, MATLAB, Infer.Net, MPlus, etc.,). The density of code underlying some algorithms makes it difficult to replicate and validate models (43). Although performance measures to compare the predictive adequacy of various machine learning techniques (area under the curve [AUC], sensitivity, specificity, positive and negative predictive values [PPV and NPV respectively], etc.,) are routinely published, studies rarely demonstrate how numeric improvements in prediction translate into better outcomes for patients. Hence, there is a pressing need for big data research to include data's relationship to improved outcomes at its core. In addition, steps need to be taken to improve the statistical literacy of healthcare professionals through greater education to bridge the divide with the big data “industry.” It is essential that clinicians embrace new findings and engage in debates surrounding big data and healthcare.

Birth cohorts have been instrumental in shedding light on asthma heterogeneity, but they alone cannot address all important questions, particularly in relation to severe disease, and the pathophysiological mechanisms underlying different phenotypes. Patient cohorts contain data which complement the information from birth cohorts, and bringing together these data assets may be essential to disaggregate asthma. Such a multi-cohort approach would enhance the credibility, reproducibility and generalizability of phenotyping results, while maximizing the benefits of accumulated and readily available evidence, but methodological challenge of how best to co-analyse the data from different contexts remains unanswered.

## The clinical utility of data-driven phenotypes

To date, numerous asthma classifications have been proposed based on observable clinical characteristics, disease severity, triggers, age of onset and inflammatory markers. For example, various atopic phenotypes (pollen sensitization with severe exacerbations, multiple allergies with severe asthma, house dust mite, multiple early/late, and late mixed inhalant) were defined based on asthma severity and allergic sensitization in pediatric populations from the TAP (44), MAAS (45), and CAPS (37) cohorts. Similarly, several inflammatory phenotypes (46) such as eosinophilic asthma, neutrophilic asthma, pauci-granulocytic asthma (24, 47–50), and Th2-high asthma (51), and trigger-induced asthma phenotypes such as cigarette smoke-induced asthma (52), air pollution-induced asthma (53), and exercise-induced asthma (54) have been identified in different populations. Although the long-term goal of the phenotype-driven approach is to broaden the personalized management of asthma, translation into clinically actionable endotypes is not readily apparent. This may, in part, be due to the limited ability to identify causative pathophysiological mechanisms of distinct subgroups of childhood asthma. The clinical utility of phenotype classification and their use in everyday clinical practice requires an improved understanding of pathophysiological mechanisms that underlie each asthma subgroup.

One way of bridging the findings from data-driven analytics into day-to-day clinical practice is by linking identified phenotypes to a specific underlying pathology, and tailoring treatment choices based on pathophysiologic mechanisms. Recent advances in molecular techniques offer promising opportunities to link phenotypes with underlying pathological mechanisms. For example, by employing machine learning, a recent study has described an architecture of multiple cytokine responses by human blood mononuclear cells to rhinovirus stimulation comprising six response profiles, and observed major differences in trajectories of asthma, allergic sensitization and lower respiratory tract infections during childhood between these profiles, suggesting that impaired anti-virus immunity may contribute to the development of a specific phenotype of troublesome childhood asthma (41). In another study, Bønnelykke et al (45). identified a novel gene (*CDHR3*) that was associated with a specific phenotype of early onset asthma with severe exacerbations. In subsequent studies, the risk variant in *CDHR3* has been reported to facilitate rhinovirus-C binding and replication (55), suggesting that the *CDHR3* may pose a risk to early-onset asthma with severe exacerbations and hospitalisations through an interaction with RV-C infection (56). Collectively, these findings highlight how the use of umbrella term “asthma” masks the complexity of disease heterogenity, and that the derivation of more precise and internally-homogenous phenotypes may be useful for providing more accurate assessment of underlying pathophysiology. Several recent studies which used machine learning-based methodologies applied to a large amount of data generated by multiplex arrays measuring IgE to more than 100 individual allergenic proteins suggest that it may be possible to develop better diagnostic algorithms to help practicing physicians differentiate between benign and clinically important allergic sensitisation to help asthma diagnosis (57–59).

In short term, the continued validation and replication of asthma phenotypes in different populations, and the integration of novel approaches such as whole genome sequencing and omics profiling to tease out pathological mechanisms underlying different phenotypes are needed to help deliver personalized medicine in clinical practice. In the longer-term, findings from large-scale data have the potential for the development of non-invasive and quick diagnostic assessments for use in clinics (57, 60).

## Future potential for refining phenotypes: integrating text mining approaches into asthma research

The exponential growth in the amount of data which is being generated in healthcare setting often makes it difficult to extract knowledge and value from a vast amount of unstructured data, or to understand whether these insights are relevant to the clinical setting (9). To date, over 119,200 scientific articles are indexed in the PubMed database under the “asthma” label, with a publication rate of more than 3,000 asthma-related papers each year (https://www.ncbi.nlm.nih.gov/pubmed/). Methodologies such as text mining are usually seen as a specialization of the broader data-mining field, with the ultimate aim of extracting useful information from unstructured data and unlocking full insight contained in huge volumes of data. They commonly rely on Natural Language Processing (NLP) methods, a key component of many Artificial Intelligence systems, dedicated to the automatic treatment of written, typed or spoken resources. The biomedical field has extended NLP solutions to biological and medical domain (also known as bioNLP) (61, 62), and demonstrated its potential use for performing extraction of asthma candidate genes (63, 64), biological and clinical concepts (65), protein-protein interactions (66), and gene-disease associations (67). Earlier applications of bioNLP in asthma research were limited to text-searching from clinical notes to characterize patients with asthma exacerbation (68), and asthma as a principal diagnosis (69). More recent studies have extended their use to include classification components of NLP which help to classify asthma status at a patient level (70).

The application of NLP to clinical problems holds out great promise of extracting biomedical relations from scientific literature and clinical narratives, and unlocking clinical information from various medical documents such as consultation notes, patient narratives or medical admission and discharge records. However, such clinical information is commonly omitted in phenotyping studies, mostly due to the unstructured nature of the data. While the integration of bioNLP methodologies with machine learning tools may help tackle the inconsistency in asthma ascertainment over many studies, one of the key limitation of bio-text mining approaches is that they still require manual curation and shared annotated datasets which are currently very limited in asthma research (71). The collaborative efforts of biomedical community toward shared objectives and tasks (72), may help overcome the current limits in BioNLP, unlock its full potential for deciphering complex disease, and provide solutions to medical problems that are too complex for a single discipline or method to resolve.

## Conclusion

Despite a significant contribution of recent phenotyping studies to our understanding of asthma heterogeneity, the translation of findings to clinical practice is hampered by a number of methodological challenges. The promise of data-driven “revolution” to support clinical decision making will not be fulfilled by technological and methodological advances alone, but by a fundamental change in medical culture, and the advancement of a team science approach (5). If we are to make a step change toward personalized medicine and capitalize on the available big data assets, we have to develop genuine cross-disciplinary collaborations, wherein data scientists who turn data into information using algorithms and machine learning, team up with medical professionals who provide deep insights on specific subjects from a clinical perspective, and prioritize which problems to solve. This may facilitate more meaningful and robust disease classification through, for example, a more informed choice of prognostic indicators, and inform the clinical decision-making process. Bringing together diverse disciplines and skill sets is a challenge for medical science in general, and complex heterogeneous long-term conditions such as asthma may offer an example ofs how targeting a particular health problem by looking at it from multiple perspectives can achieve insights that translate to patient benefit through the delivery of personalized medicine.

## Author contributions

AC, CO, and SH conceived the idea; SF and CF provided input on the methodology; All authors wrote the report.

### Conflict of interest statement

The authors declare that the research was conducted in the absence of any commercial or financial relationships that could be construed as a potential conflict of interest.

## Footnotes

^1^http://www.propellerhealth.com
